# Experts' attitudes towards medical futility: an empirical survey from Japan

**DOI:** 10.1186/1472-6939-7-8

**Published:** 2006-06-10

**Authors:** Alireza Bagheri, Atsushi Asai, Ryuichi Ida

**Affiliations:** 1 Graduate School of Law, Kyoto University, Yoshida, Sakyo-ku, 606-8501 Kyoto, Japan; 2 Department of Bioethics, Faculty of Medical and Pharmaceutical Science, Kumamoto University, 1-1-1 Honjo, 860-8556 Kumamoto, Japan

## Abstract

**Background:**

The current debate about medical futility is mostly driven by theoretical and personal perspectives and there is a lack of empirical data to document experts and public attitudes towards medical futility.

**Methods:**

To examine the attitudes of the Japanese experts in the fields relevant to medical futility a questionnaire survey was conducted among the members of the Japan Association for Bioethics. A total number of 108 questionnaires returned filled in, giving a response rate of 50.9%. Among the respondents 62% were healthcare professionals (HCPs) and 37% were non-healthcare professionals (Non-HCPs).

**Results:**

The majority of respondents (67.6 %) believed that a physician's refusal to provide or continue a treatment on the ground of futility judgment could never be morally justified but 22.2% approved such refusal with conditions. In the case of physiologically futile care, three-quarters believed that a physician should inform the patient/family of his futility judgment and it would be the patient who could decide what should be done next, based on his/her value judgment. However more than 10% said that a physician should ask about a patient's value and goals, but the final decision was left to the doctor not the patient. There was no statistically significant difference between HCPs and Non-HCPs (*p *= 0.676). Of respondents 67.6% believed that practical guidelines set up by the health authority would be helpful in futility judgment.

**Conclusion:**

The results show that there is no support for the physicians' unilateral decision- making on futile care. This survey highlights medical futility as an emerging issue in Japanese healthcare and emphasizes on the need for public discussion and policy development.

## Background

In the industrialized nations facing an ageing population and escalating healthcare costs, the issue of medical futility has become the object of extended critical attention especially among medical doctors, bioethicists and health policy makers.

In particular, the care of the elderly is going to be a major health problem in more developed regions of the world like Japan and Western countries. These concerns have led to a call for a re-approach to the matter of medical futility. The question however is the appropriate use of technology at the end of life. Issues have also arisen regarding the quality of life, medical procedure with sometimes only marginal benefits to the patients and the burdens that medical technology imposes on patients, families, and the society. The impact of the futility concept on decision-making has been hampered by a lack of a clear definition of medical futility itself. The current debate about the issue of medical futility is mostly driven by theoretical and limited personal assumptions. There is a lack of empirical data to document experts and public attitudes towards medical futility.

Like many other countries, in Japan too the issue of medical futility is relatively new to the health care system and there is a lack of practical frameworks or regulations to deal with the issue. This paper presents empirical data about Japanese experts' attitudes towards medical futility and its relevance and application in Japanese healthcare system.

### The healthcare system and end of life decision making in Japan

Japan with more than 126 million habitants has emerged as an industrial country after World War II. Currently life expectancy at birth is 78.64 for men and 85.59 for women and the population of Japanese over 90 years old is 1,016,000 and is estimated to become 3,149,000 by 2029. The number one cause of death is malignant neoplasms in both sexes [1]. The important feature is that the nature of such diseases makes the end of life period longer, more burdensome and costly for the family and the society alike.

The average length of hospitalizations in Japan, excluding those for special cases such as psychiatric illness, is about 45.4 days, which is much longer than in most Western nations. For the elderly, hospitals often assume the function of nursing homes [2].

There is universal health insurance, which supports the concept of social justice and access for all to health care in Japan. Aged people are also expected to pay a slight proportion of the medical costs themselves. It is noteworthy to mention that the post-World War II baby-boom generation begins turning sixty years old in 2007. There are concerns in the Japanese society, and among health policy makers about how they can cope with the new situation. For instance the effect of fee-for-service payment for long-term care has been cited as leading to over-treatment [3]. In order to accommodate ever increasing medical costs, the Japanese government has taken initiatives to reform the medical system.

With the anticipated increase of the healthcare costs in an ageing population which is rapidly growing, ongoing changes in national health insurance, pension, and mass retirement of nearly nine percent of the nation's workforce in Japan, the issue of medical futility is going to appear as an emerging issue in years ahead and hit the ethical and health policy debates. Japanese traditional culture, the role of family, physicians' strong authority and the current socio-economical situation, all have a great influence in shaping the health care system. Many commentators have demonstrated the important role of the family in Japanese society and family decision-making on behalf of the patient is generally an accepted behavior. The process of dying is regarded not as an individual event but as a family event in the Japanese culture [4]. However the end of life decision making policy is under criticism. For instance it has been claimed that the problem in the end of life issues is not lack of resources but too much attention to the goals of sustaining life, without enough attention being given to the wishes of the patient as a person [5]. Regarding physicians' authority in clinical settings, Hamano refers to the Japanese health care as a paternalistic medical system and argues that because of doctors' paternalism and lack of sufficient communication between doctors and patients, individuals are under pressure for end of life decision making [6].

## Methods

The respondents' attitudes towards medical futility were assessed by conducting a questionnaire survey. A pilot study has been done to develop a 20-item questionnaire in Japanese. The final questionnaire was handed out to 212 members of the Japanese Association for Bioethics (JAB) during the 21^st ^annual meeting in November 2005. General definitions of terminology were provided in the questionnaire sheet [7,8] as follow:

a) Quantitative (physiologic) futility: if a treatment in 100 consecutive previous cases is seen to be futile, then sufficient quantitative evidence exists to declare that this treatment is futile in a current case (the treatment is medically ineffective because it would not work).

b) Qualitative (evaluative) futility: if a treatment could not result in patient's discharge from the hospital independently (the treatment is inappropriate because it would just not be worth it).

The data was finally analyzed using a statistical package for social sciences, SPSS version 13.0.

## Results

A total number of 108 responses returned via a return postage-paid envelope, giving a response rate of 50.9%. As the profile of the respondents shows in table 1, of respondents 62% were health care professionals, 37% were non-healthcare professionals. Of the respondents, 41% were female and 57 % male.

**Table 1:** Profile of respondents to medical futility survey

		**n (%)**	**Missing**
**Sex**			3 (2.8%)
	Male	61 (56.5%)	
	Female	44 (40.7%)	
**Age**			3 (2.8%)
	≥ 55 y/o	36 (33.3%)	
	35≤ y/o<55	51 (47.2%)	
	<35 y/o	18 (16.7%)	
**Background**			1 (0.9%)
	HCPs	67 (62%)	
	Non- HCPs	40 (37%)	

Healthcare professionals (HCPs), Non-healthcare professionals (Non-HCPs)

### Attitudes towards medical futility in general

The majority (67.6%) of respondents believed that physicians' refusal to offer or continue a treatment on the ground of futility judgment can never be morally justified but 22.2% approved such refusal with conditions. Of them 66.7% said "it can be justified if the demanded treatment is physiologically futile" and 12.5% said "if care can be provided to another patient who has a more just claim on the scarce resources". As the table 3 shows, there were no statistical significance differences between HCPs and Non-HCPs. Although a higher proportion of healthcare professionals (73%) disagree with the morality of such refusal based on medical futility, compare to non-healthcare professionals (60%), the difference did not have statistical significance (*p *= 0.252, *x*^*2 *^= 1.315).

Asking how to evaluate a futile case, almost two-third responded that it should be evaluated based on the doctor's medical judgment and the patient's value judgment. However 19.5% said that it should be decided based on a pure medical judgment, and only one person said it should be based on a doctor's own medical and value judgment.

Of the respondents 28.7% said professional judgment never allows physicians to dictate the care of the patient, but more than 31% said it allows if it is not in conflict with patient's value. However almost fourteen percent said in case of a pure physiologic futility a doctor could decide about the treatment by him or herself.

Regarding the important factors in futility judgment in a multiple choice question, 77.8% marked "patient/family wishes" and 63.9% and 45.4% marked "availability of resources" and "patient's age", respectively.

Experts were asked to rank how disagreement between physician and patient/family about ongoing treatment should be solved in a multiple choice question. The option "physician should try to convince patient/family by providing more information", was the first choice for 67.6% of respondents with no significant difference between health care and non-healthcare professionals (*p *= 0.175). The other option of "resolving the issue by hospital ethics committee" was the first choice for 9.3% of respondents. The least acceptable solution was "taking the case to the court" as a first option, marked by only one respondent.

Regarding decision making about the persistent vegetative state (PVS) in a society with scarce health resources, 58.3% preferred a shared decision-making by physician and patient/family. While almost 18% preferred decision making using guidelines set by the health authority, six people marked decision making based on guidelines set by the hospitals. Eight respondents were in favor of decision making only by the patient's family and interestingly nobody was agreed with physician's unilateral decision-making in case of PVS patient.

Whether practical guidelines by the health authorities can help physicians in decision- making on futile care, almost 68% of respondents believed it could help and ten persons disagreed. However 21.3% said it is uncertain to achieve such a goal by guidelines development.

### Attitudes towards physiologic and evaluative futility

It was asked, if in physician's judgment a treatment is physiologically futile, is there any obligation on physician to inform the patient or family? The majority (85.2%) affirmed such obligation. Among those who affirmed, 91.2% believed that doctor should also let the patient to decide about the treatment, but 8.8% believed that still it is the doctor who should decide what to do. As the table 3 shows there was no difference between HCPs and Non-HCPs, as more than 86% of both groups believed physicians must inform the patient and let them decide about the course of treatment (*p *= *0.844*, *x*^*2 *^= *.039*). However eight percent of the respondents disagreed and said it is not necessary to communicate the issue with patient and her or his family.

Respondents were asked a control question regarding physiologic futility based on physician's judgment, with different wording, the answers to both were comparable, as a three-quarter believed that physician should inform the patient/family about his futility judgment and it is the patient who can decide what has to be done next based on his/her value judgment. Only one respondent believed that unilateral decision-making by doctors is the solution. However more than 10% said that physicians should ask about patient's value and goals, but the final decision is left to the doctor not the patient. There was no statistical significant difference between HCPs and Non-HCPs (*p *= 0.676).

Replying to the question "if the family still wants everything done in case of physician's physiologic futility judgment, what is the next step?", more than 24% replied that patient and family wishes had to be followed. Half of the respondents believed that physician should try to convince the patient/family about his judgment by negotiation. Only two respondents believed that physician should be authorized for a unilateral decision- making. Five respondents said that the next step was to transfer the patient to another doctor. Of the respondents 46% believed that if professional judgments differ from patient/family's values, physicians should compromise their value and the priority should be given to patient's value. Interestingly in choosing this option, there was a significant difference (*p *= 0.028) between respondents age over 55 years old (82.6%) and respondents younger than 55 years old (100%). Only 4 respondents believed that physicians should rely on their own values because professional judgment had a privilege. The written comments include: Continue discussions to reach a mutual understanding (twenty five responses), the ethics committee involvement (three responses), asking for second opinion (two responses), respecting patients' wishes (four responses).

As table 2 shows, 87% of respondents believed that physicians' professional judgment would not be a "value free" judgment. When a hypothetical case was presented in which the treatment would likely achieve the patient's goals, but the clinician perceived those goals to be valueless, almost 70% replied: they did not believe that physicians had a legitimate role in blocking access to the treatment. However 13% believed so. Asked whether physicians should be allowed to judge about evaluative futility and rely on their own personal beliefs and values? Almost 65% believed that physician could only judge on physiologic futility and "value judgment" had to be left to the patients. In contrary 28.7% said yes, because medical judgment is always mixed with value judgment. However, as the table 3 shows there was no significant difference between HCP and Non-HCP (*p *= 0.648) and also no significant difference between male and female participants in this survey (*p *= 0.463). In written comments for this question some respondents have authorized physicians to judge about evaluative futility in the following situations: in an emergency case which patient's life is in danger (seven responses); in case of incompetent patients when no family is available (two responses); and finally when the patient leave all decisions to the physician (two responses).

**Table 2:** The role of physicians in decision making about futile care

**Questions**	**Yes**	**No**	Comments*	**Missing**
If treatment is physiologically futile, is there any obligation on physician to inform the patient?	85.2% (92)	7.4% (8)	5.6% (6)	1.9% (2)
Should physicians' professional judgment be "value free"?	6.5% (7)	87% (94)	4.6% (5)	1.9% (2)
Do you think that physicians have a legitimate role in blocking access to the treatment even if it would likely achieve the patient's goals?	13% (14)	69.4% (75)	13% (14)	4.6% (5)
Should physicians be allowed to judge about evaluative futility and rely on their own personal beliefs and values?	28.7% (31)	64.8% (70)	-------	6.5% (7)
Should physicians be authorized to refuse a treatment which works but because of the patient's poor prognosis he believes that it is not worth trying?	25% (27)	55.6% (60)	17.6% (19)	1.9% (2)
Should physicians be empowered to impose evaluative judgment that conforms to professional standards and emerging societal interest?	4.6% (5)	75% (81)	17.6% (19)	2.8% (3)

* In some questions, an option was to write their own comments.

**Table 3:** Comparison between HCPs and Non-HCPs.

**Question**	**HCP**	**Non-HCP**	***P *Value**
*Overall*	*67 (62%)*	*40 (37%)*2.	
Is a physician's refusal to provide or continue treatment on the ground of futility judgment morally sound?			
Yes	13(19.4%)	11(27.5%)	
No	49(73.1%)	24(60%)	0.252
Others (Comments)	5(7.5%)	5(12.5%)	----

If treatment is physiologically futile, is there any obligation on a physician to inform the patient?			
Yes	56(86.2)	35(87.5%)	
No/Others	9(13.9%)	5(12.5%)	0.884

Should physicians be allowed to judge about evaluative futility and rely on their own personal beliefs and values?			
Yes	18(29%)	13(33.3%)	
No	44(71%)	26(66.7%)	0.648

* Written comments were excluded in the analysis.

As table 2 shows 55.6% of respondents believed when an aggressive treatment works, physicians should not be authorized to refuse the treatment even if they believe it is not worth trying because of the patient's poor prognosis. However, one-fourth of the respondents have authorized physician to do so.

The question was asked whether in their opinion decision based on value judgment can be considered as patient's right, 67.6% said no, and 16.7% replied yes, more than 11% marked "I do not know".

In a multiple choices question as "what would be the consequence of granting physicians wide latitude in formulating and imposing their own personal value (evaluative) judgments", more than 70% said it would be a paternalistic medical system. Almost 60% said it would cause mistrust of medical professions. However four respondents said it would provide better health for the patients.

As the table 2, shows when asked whether physicians should be empowered to impose evaluative judgment that conformed to professional standards and emerging societal norms and interest, a three-quarter said no, because nobody can deprive patients from their rights to health services. Only five people said yes, because physicians are responsible for spending health resources. Nineteen, gave their comments such as: the ethics committee should intervene, brain death should be considered an exception, and information disclosure is necessary.

### Medical futility and Japanese situation

In this survey we have asked two questions which were directly related to the Japanese health care system. Replying to the question whether "medical futility" is related to Japanese healthcare, almost 64% of respondents believed that medical futility was an especially relevant issue to Japan. One-fourth thought that because of the growing elderly population it would be an emerging issue in future. However two persons believed that medical futility was not relevant to Japanese healthcare.

We also asked the question: "given the fact that currently in Japan there is enough financial support and health insurance coverage; do you think that an aggressive treatment should never be stopped on the ground of medical futility?" Twenty four percent of respondents agreed but 56.5% disagreed and believed that despite a good financial support still the treatment could be stopped. Comments include (each, one response): In Japan, financial support still is not sufficient; treatments might be stopped if public support limited; the treatment should never be stopped solely based on futility; we should not relate financial problems to the question of life; consensus should be developed by the third party; if aggressive treatment inflicted pain on the patient, then it should be stopped and passive treatment should take over.

## Discussion

Although the size of our study's target group prevents us from drawing definitive conclusions about the Japanese healthcare system, a few observations are worth highlighting as possible leads for further research and directions for improvement.

As mentioned earlier the current debate on medical futility is mostly based on the theoretical assumptions and there has been no similar empirical study either in Japan or elsewhere to compare with the results of our study.

Japanese traditional and contemporary views of life and death derive mainly from Shinto and Japanese Buddhism. The latter has a great impact on the end of life decision making.

While the "dying process" is important in Shintoism, "reincarnation" has received more attention in Buddhism. As Noritoshi Tanida points out regarding end of life decision making in contemporary Japan, the prevailing practice is "prolongation of the dying process" [9]. In our survey the majority of respondents were in favor of following patients' and family wishes which in many cases are prolongation of life. They have also emphasized on establishing a dialogue between physicians, patients and their family to reach an agreement.

While ethics consultations were found helpful in resolving the conflicts over treatment decisions [10], the results in our survey don't strongly support a role in solving disagreement on futility judgment for the hospital ethics committees. In Japan hospital ethics committees are not well established. A study shows that more than 75% of the hospitals in Japan have no ethics committee [11]. This might be one of the reasons why our respondents rarely mentioned about the role of ethics committees in solving the conflict over futility judgment. Interestingly the least acceptable solution of a disagreement between physician and patient/family about ongoing treatment was taking the case to the court.

Despite the widespread practice and a general idea that in case of physiologically futile treatment the HCPs should be able to refuse the treatment, the majority of respondents believed that even in case of physiologic futility the decision-making have to be left to the patients and their family. In case of evaluative futility also, they did not authorized physicians to withhold the treatment.

In our survey there was a strong support for the shared decision-making process involving physicians, patients and their family, and in case of disagreement respondents believed that physicians should compromise their own values and the priority should be given to patient's wishes. Whether the reason behind this finding as Campbell and Ikegami points out is that: "in Japan physicians and hospitals must strive to keep patients satisfied because provider income depends on volume", needs more investigations [12]. Although many commentators have referred to Japanese healthcare system as a paternalistic medical system, which physicians have more power to dictate the treatment, in our survey there was no support for physician's unilateral decision-making. The respondents expressed their concern about the consequences of granting physicians wide latitude in formulating medical futility based on their own values, and called it "paternalism". They believe that it may cause more mistrust of medical professions. However whether our result support the idea that Japanese medical professions are transforming from a paternalistic to non-paternalism (or an individualistic one), or to claim that the final decision-making authority is with patients and family as a widely accepted practice amongst Japanese HCPs, requires another study with the participation of HCPs who are not directly involved in bioethical discussions. Again, acknowledging the limited data, we draw attention that the participants in this survey were medical and non-medical professionals involved in bioethical discussions and are unlikely representative of the Japanese HCPs. Therefore the authors can not generalize the survey's results for all Japanese medical professions.

In Japan, end of life decision-making is more family-oriented as Seishi Fukuma says; whether life-prolonging treatment to be administered, physicians ask the family and more often not little consideration is given to the opinion of the elderly persons themselves [13].

However the experience of legislation on brain death and organ transplantation in Japan shows that in any policy making for end of life issues, how to include the role of family in balance with the individual autonomy is very crucial [14].

As it has been shown in a survey in Canada, physicians were cited to provide futile care because of prognostic uncertainty (84%) and legal pressures (75%), [15]. In Japan also it seems that the impact of some court cases such as the recent case in the Hokkaido Haboro hospital influences physicians' attitudes towards futile treatment. In February 14, 2004, a 90 years old patient with cardiac arrest has been put under the ventilator after cardiopulmonary resuscitation. The next day, respirator was removed from the patient by the attending doctor after convincing the family that continuing the treatment is futile. But in April, 2005, the Hokkaido police filed a lawsuit against doctor-in-charge, stating that withdrawal of life sustaining treatment was an act of murder by the doctor (Yomiuri Newspaper, May 19, 2005). Although the Hokkaido attorney office decided not to prosecute for murder, according to the media report, police involvement and being questioned by the police is sufficient enough to make doctors to think that stopping life sustaining treatment is not only immoral but also illegal.

Asai and Onishi pointed out some years ago that in Japan, societal interest and scarcity of resources are not big concerns and withholding treatment for a particular patient in consideration of financial or societal interest is rare in the clinical decision making [16]. Our results show a slight change in that direction; concerns on health expenditures and financial health care support are growing and many of the respondents believe that medical futility is going to be especially relevant to Japanese health care system.

## Conclusion

In a constructive approach to the issue of medical futility, both theoretical and empirical researches are crucial and neither could fulfill it alone. The synergy of theoretical discussions and empirical data would offer policy makers appropriate tools to adopt more accurate and efficient policies for medical futility in clinical settings.

The majority believe that in case of physiologic futility physician has to inform the patient/family about his futility judgment and decision-making should be left to patients and family. There was no support for the physicians' unilateral decision-making about futile care and the respondents were in favor of a shared decision-making. 

Discussions about medical futility are relatively new to Japanese health care and along with ongoing reform of the medical system, health insurance policies and an increasing aging population, discussion about the application of life sustaining treatment in marginal benefit and medical futility are going to be more important in Japan. There was more support for developing national guidelines on dealing with medical futility rather than guidelines set up by local hospitals. The legislative experience of brain death and organ transplantation in Japan shows that an important issue in policy development on medical futility would be how to include the role of family. However a rational approach to the role of family in Japanese society based on current cultural changes will be the key issue to success or fail of such regulatory attempts.

## Competing interests

The author(s) declare that they have no competing interests.

## Authors' contributions

Alireza Bagheri contributed substantially to the conception and design, analysis and interpretation of data, drafting the article and revising it critically and gave final approval for the version to be published.

Atsushi Asai contributed substantially to the analysis and interpretation of data and revising it critically and gave final approval for the version to be published.

Ryuichi Ida contributed substantially to the conception and design, interpretation of data, revising it critically and gave final approval for the version to be published.

## Pre-publication history

The pre-publication history for this paper can be accessed here:


